# The Efficacy of Different Material Scaffold-Guided Cell Transplantation in the Treatment of Spinal Cord Injury in Rats: A Systematic Review and Network Meta-analysis

**DOI:** 10.1007/s10571-024-01465-6

**Published:** 2024-05-04

**Authors:** Zhihua Wang, Jun Li, Tianqi Xu, Boyu Guo, Zhiping Xie, Meihua Li

**Affiliations:** 1 Department of Neurosurgery, The First Affiliated Hospital of Nanchang University, No.17, Yongwai Street, Nanchang, 330006 Jiangxi Province China; 2 Postdoctoral Innovation Practice Base, The First Affiliated Hospital of Nanchang University, Nanchang, Jiangxi Province China; 3 Department of the Second Clinical Medical College of Nanchang University, No.460, BaYi Street, Nanchang, 330006 Jiangxi Province China; 4 Department of the First Clinical Medical College of Nanchang University, No.460, BaYi Street, Nanchang, 330006 Jiangxi Province China; 5 Department of Neurosurgery, Jiangxi Provincial People’s Hospital, The First Affiliated Hospital of Nanchang Medical College, No.152 Aiguo Road, Nanchang, 330006 Jiangxi Province China; 6 Department of Neurosurgery, Xiangya Hospital Jiangxi Hospital, Central South University, Nanchang, Jiangxi Province China

**Keywords:** Spinal cord injury, Scaffolds, Cell transplantation, Meta-analysis

## Abstract

**Abstract:**

Cell transplantation is a promising treatment option for spinal cord injury (SCI). However, there is no consensus on the choice of carrier scaffolds to host the cells. This study aims to evaluate the efficacy of different material scaffold-mediated cell transplantation in treating SCI in rats. According to PRISMA’s principle, Embase, PubMed, Web of Science, and Cochrane databases were searched, and relevant literature was referenced. Only original research on cell transplantation plus natural or synthetic scaffolds in SCI rats was included. Direct and indirect evidence for improving hind limb motor function was pooled through meta-analysis. A subgroup analysis of some factors that may affect the therapeutic effect was conducted to understand the results fully. In total, 25 studies met the inclusion criteria, in which 293 rats received sham surgery, 78 rats received synthetic material scaffolds, and 219 rats received natural materials scaffolds. The network meta-analysis demonstrated that although synthetic scaffolds were slightly inferior to natural scaffolds in terms of restoring motor function in cell transplantation of SCI rats, no statistical differences were observed between the two (MD: −0.35; 95% CI −2.6 to 1.9). Moreover, the subgroup analysis revealed that the type and number of cells may be important factors in therapeutic efficacy (*P* < 0.01). Natural scaffolds and synthetic scaffolds are equally effective in cell transplantation of SCI rats without significant differences. In the future, the findings need to be validated in multicenter, large-scale, randomized controlled trials in clinical practice.

*Trial registration*: Registration ID CRD42024459674 (PROSPERO).

**Graphical Abstract:**

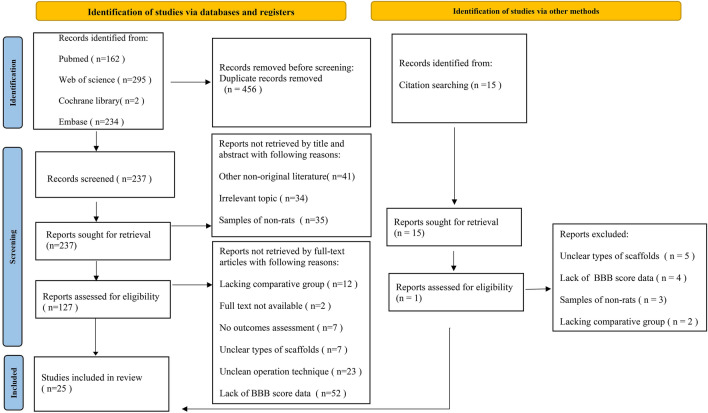

**Supplementary Information:**

The online version contains supplementary material available at 10.1007/s10571-024-01465-6.

## Introduction

Spinal cord injury (SCI) is a severe neurological disorder usually caused by mechanical trauma, such as traffic accidents, falls, and sports-related injuries. Not only can it lead to a series of symptoms, such as severe loss of movement, sensory impairment, and autonomic nervous dysfunction, but it also causes complications, such as neuropathic pain, that seriously impair the patient’s quality of life. SCI is estimated to affect nearly 180,000 people each year due to its high incidence, disability, and death rate (Fitzharris et al. 2014). Currently, the clinical treatment of SCI primarily includes decompression surgery, steroid hormone shock therapy, and neurological rehabilitation exercise (Silva et al. 2014). The above-mentioned treatments alleviated the secondary neuronal degeneration and necrosis to a certain extent. However, due to the highly differentiated and nonrenewable nature of neurons, direct primary neuronal death often makes the prognosis of patients unsatisfactory (Badhiwala et al. 2021; Curt et al. 2008). Theoretically, exogenously restoring neurons is the optimal approach to SCI; thus, cell transplantation (mature nerve cells or stem cells with the potential to differentiate) has emerged as a treatment for SCI (Tetzlaff et al. 2011).

Several preclinical studies demonstrated that stem cells transplanted in SCI animal models could restore synaptic connections and neural networks through direct neuronal regeneration or paracrine effects (Rong et al. 2019; Li et al. 2020; Deng et al. 2015). Some problems still need to be addressed in the large-scale clinical application of cell transplantation in patients with SCI, such as determining the best timing for neural stem cell therapy, the source of neural stem cells, the route of administration of cell transplantation, the number of transplanted cells, and the best combination of treatment. However, advancements in stem cell therapy technology and tissue engineering technology are continuously unlocking the potential of cell transplantation. Therefore, studies should be conducted to comprehensively evaluate and analyze the efficacy of cell transplantation in SCI. Some reports have shown the efficacy of single-cell transplantation in the subacute phase (Ide et al. 2010; Yang et al. 2020) and some reports have started clinical trials (Anderson et al. 2017; Karamouzian et al. 2012; Sugai et al. 2021). In order to achieve the ideal recovery effect of cell transplantation therapy, the emergence of scaffolds has enriched the treatment of cell transplantation. At present, the curative effects of SCI may not be significantly improved through single-cell transplantation, which may be associated with the survival of transplanted cells, directional migration to the lesion site, differentiation, and axonal regeneration of transplanted cells (Yousefifard et al. 2022; Yousefifard et al. 2019). Comprehensive cell transplantation therapy combined with tissue engineering scaffolds became a trend. Scaffolds can fill the gap between lesion sites and serve as a supportive physical medium. Furthermore, they can also preserve the activity of transplanted cells and mediate the directional growth of axons to act as a bridge to restore neural networks. The use of biomaterials can enhance the effect of cell transplantation. In future, scaffold and cell engineering technologies may improve cell survival, integration, and therapeutic efficiency (Zipser et al. 2022). Compared with single-cell transplantation, the therapeutic effect of cell transplantation with scaffold combined therapy is synergistically enhanced, which is more effective in promoting neurological recovery after SCI (Zweckberger et al. 2016; Liu et al. 2013a, b, c). Consequently, many studies on scaffolds combined with cell transplantation were conducted in animal models to pave the way for clinical practice.

Scaffolds are divided into natural biomaterials (such as hyaluronic acid, collagen, and acellular scaffolds) and synthetic biomaterials (polylactic acid-glycolic acid copolymer and polylactic acid), both of which have their advantages (Wang et al. 2015; Kubinová and Syková 2010). The primary advantages of natural biomaterials are their excellent biocompatibility, low immunogenicity, and nontoxic degradation (Libro et al. 2017). Synthetic biomaterials are usually easier to adjust than natural biomaterials. For instance, the porosity, stiffness, and degradation rate of synthetic biomaterials can be altered to match different types of tissue (Subramanian et al. 2009). Concurrently, they also have a low incidence of inflammatory response (Subramanian et al. 2009). Most prior studies on cell transplantation for SCI focused on stem cells or nerve cells in cell transplantation (Antonic et al. 2013), but no systematic review on the selection of scaffolds was exclusively conducted. Therefore, analyzing the therapeutic effects of scaffold-assisted cell transplantation in SCI is necessary, as this will provide a direction for subsequent large-scale clinical trials and applications of patients.

## Methods

### Literature Search

This meta-analysis was conducted under the requirements listed in the Preferred Reporting Items of Systematic Review and Meta-Analysis (PRISMA). English articles of randomized controlled trials (RCTs) in EMBASE, PubMed, Web of Science, and Cochrane databases were searched until September 2023. Moreover, if the article that met our criteria appeared in its references, the full text from the PubMed database was retrieved to evaluate its suitability for our study. The subject search terms were as follows in all four databases: Spinal Cord Injury AND (“Cell Transplantation” OR “Stem Cell Transplantation”) AND (“Scaffolds” OR “Tissue Scaffolds”). More details could be seen in Supplementary material.

### Selection Criteria

After removing duplicate references retrieved from the above databases  and sorting out all references, the inclusion criteria are as follows:

1. The animal model of SCI is the rat.
2. SCI modeling, such as contusion, hemisection, or transection, must be accepted by the mainstream.
3. The components of the scaffold used for cell transplantation are clear and detailed.
4. There must be a blank control (SCI group without therapeutic measures). Vehicle group: the rats were injected intrathecal saline or PBS equal to the amount of cell fluid after surgery of causing SCI.
5. The follow-up time is more than four weeks, and there are outcome measures to evaluate the motor function.
6. The articles are published in English.

The exclusion criteria are as follows:

1. Non-English literature and review articles are excluded.
2. No control group is compared.
3. The postoperative motor function of rats is not quantitatively reported.

### Literature Collection

Two researchers (Jun Li and Zhihua Wang) independently searched for relevant literature from various electronic literature databases and selected all studies that met the above criteria through titles and abstracts (checking the full text if necessary). Both researchers were responsible for assessing whether a manuscript was suitable for inclusion in the meta-analysis. If the two had opposing viewpoints, they would resolve them through group discussion and negotiation until a consensus was reached.

### Data Extraction

Two investigators (Zhiping Xie and Tianqi Xu) independently extracted the main data of every article from the selected original researches. The data included (1) basic information about every article, such as the first author and year of publication of every article; (2) detailed information, such as gender and weight of rats, the corresponding number of rats in each group, method of inducing spinal cord injury (contusion, spinal cord hemisection, or transection), cell donor and type, transplanted cell quantity, scaffold material, use of antibiotics, use of immunosuppressants, the time interval from SCI to corresponding transplantation treatment, the duration of prognosis observation, motor function score, and double-blind assessment of motor function. All included literature used Basso–Beattie–Bresnahan (BBB) scores to evaluate the motor function of the hind limbs of rats, and the scores were positively proportional to the recovery of the motor function of rats (Basso et al. 1995). We preferred using digital data reported in the articles. If the data were presented only in the form of charts, PLOT digital software was used to extract data. If necessary, we would contact the corresponding authors of eligible studies via e-mail to obtain sufficient data.

### Quality Assessment of Literature

The quality of the included studies was independently evaluated by two authors (Boyu Guo and Meihua Li). Because all included studies were RCTs on rats, Hassannejad et al.’s study was reasonably selected to make an overall judgment on the quality of each study (Hassannejad et al. 2016). They conducted a risk assessment using a 15-item checklist as a guide. They assessed risks from the animal (4 items), assessment (10 items), and housing (1 item) variables for evaluating the quality of the included studies (Table 1). The two authors strictly adhered to the research standards established by Hassannejad et al. If their opinions on a study differed, the group would discuss it until they agreed.

**Table 1:** Quality assessment of included studies

Author (year)	Item 1	Item 2	Item 3	Item 4	Item 5	Item 6	Item 7	Item 8	Item 9	Item 10	Item 11	Item 12	Item 13	Item 14	Item 15
Wang (2020)	L	L	L	L	L	L	L	L	L	L	L	L	L	L	U
Wang (2017)	L	L	L	L	L	L	L	L	L	L	L	L	L	L	U
Kim (2016)	L	L	L	L	L	L	L	L	L	L	L	L	L	L	U
You (2019)	L	L	L	L	L	L	L	L	L	L	U	L	L	L	U
Liu (2022)	L	L	L	L	L	L	L	L	L	L	U	L	L	L	U
Hosseini (2016)	L	L	L	L	L	L	L	L	L	L	L	L	L	L	U
Wang (2016)	L	L	L	L	L	L	L	L	L	L	L	U	L	L	U
Jiao (2017)	L	L	L	L	L	L	L	L	L	L	U	U	L	L	U
Peng (2018)	L	L	L	L	L	L	L	L	L	L	L	L	L	L	L
Chen (2014)	L	L	L	L	L	L	L	L	L	L	L	L	L	L	U
Liu (2013a)	L	L	L	L	L	L	L	L	L	L	L	L	L	L	U
Liu (2013b)	L	L	L	L	L	L	L	L	L	L	L	L	L	L	L
Marchini (2019)	L	L	L	L	L	L	L	L	L	L	U	U	L	L	L
Zaminy (2013)	L	L	L	L	L	L	L	L	L	L	U	U	L	L	U
Zarei-Kheirabadi (2020)	L	L	L	L	L	L	L	L	L	L	L	L	L	L	U
Hatami (2009)	L	L	L	L	L	L	L	L	L	L	U	L	L	L	L
Wang (2021)	L	L	L	L	L	L	L	L	L	L	L	L	L	L	U
Tavakol (2014)	L	L	L	L	L	L	L	L	L	L	L	L	L	L	L
Deng (2020)	L	L	L	L	L	L	L	L	L	L	L	L	L	L	U
Raynald (2019)	L	L	L	L	L	L	L	L	L	L	L	L	L	L	U
Yang (2017)	L	L	L	L	L	L	L	L	L	L	L	L	L	L	U
Han (2019)	L	L	L	L	L	L	L	L	L	L	L	L	L	L	U
Ropper (2016)	L	L	L	L	L	L	L	L	L	L	L	L	L	L	U
Liu (2015)	L	L	L	L	L	L	L	L	L	L	L	L	L	L	U
Hejĉl (2010)	L	L	L	L	L	L	L	L	L	L	L	L	L	L	L

L: indicates low risk of bias; U: the prescience of risk of bias is unclear due to insufficient descriptions in the article; H: indicates high risk of bias

1. Species; 2. Using appropriate tests; 3. Severity of injury; 4. Level of injury; 5. Age/weight; 6. Number of animals per group; 7. Designation of strain; 8. Definition of control; 9. Description of statistical analysis; 10. Regulation and ethics; 11. Bladder expression; 12. Blindness of assessor; 13. Genetic background; 14. Method of allocation to treatments; 15. Description of the reasons to exclude animals from the experiment during the study (attrition)

### Statistical Analysis

All statistical analyses were conducted using R software (version 5.4.1, Cochrane Collaboration, London). Since the outcome measures of all included studies were presented in the form of continuous data (BBB score), we used Mean ± SD to directly or indirectly compare the therapeutic effects of cell transplantation plus scaffolds of different materials in rats with SCI. As the meta-analysis included different studies and there was relatively significant heterogeneity between studies (*P* < 0.1 or *I*^2^ > 50%), the data were consolidated using a random-effects model. Furthermore, subgroup analyses were performed to trace the source of heterogeneity or the study was subdivided to get a full picture of the results. Published bias was examined using a funnel plot. If the funnel plot was visually symmetric, this indicates that there was no publication bias.

## Results

### The Characteristics of Included Studies

**Fig. 1:**
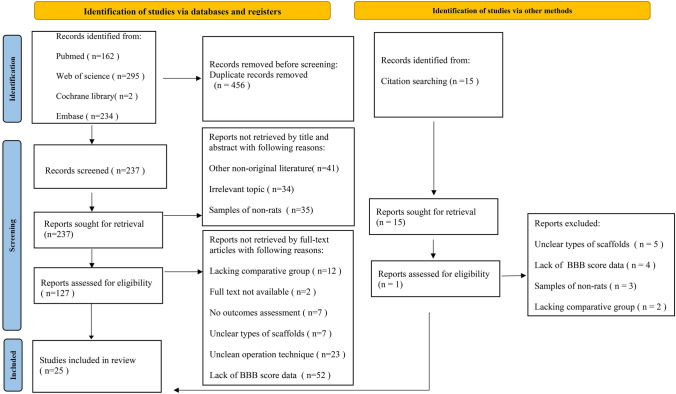
The PRISMA flow diagram of the network meta-analysis

After four databases or interesting citations were comprehensively searched and duplicate references were eliminated, 252 articles were reviewed by title and abstract. After preliminary screening, the full text of 142 articles was reviewed to assess the necessity of inclusion. Finally, 25 articles were included in the network meta-analysis (NMA) (Chen et al. 2014; Deng et al. 2020; Han et al. 2019; Hatami et al. 2009; Hejcl et al. 2010; Hosseini et al. 2016; Jiao et al. 2017; Kim et al. 2016; Liu et al. 2015; Liu et al. 2013a; Liu et al. 2013b; Liu et al. 2022; Marchini et al. 2019; Peng et al. 2018; Raynald et al. 2019; Ropper et al. 2017; Tavakol et al. 2014; Wang et al. 2020; Wang et al. 2016; Wang et al. 2021; Wang et al. 2017; Yang et al. 2017; You et al. 2019; Zaminy et al. 2013;Zarei-Kheirabadi et al. 2020). Figure 1 demonstrates the details of the inclusion assessment.

Of the 25 articles, 24 were two-arm studies, i.e., cell transplantation + scaffolds (natural or synthetic materials) vs. vehicle control group, except for one study that was a three-arm study directly comparing both different types of scaffolds to the control group (Kim et al. 2016). In other words, while nineteen studies compared the efficacy differences between cell transplantation + natural scaffolds in SCI rats and vehicle control group in SCI rats, seven articles focused on synthetic scaffolds (Han et al. 2019; Hejcl et al. 2010; Kim et al. 2016; Liu et al. 2015; Raynald et al. 2019; Ropper et al. 2017; Yang et al. 2017).

A total of 590 SCI rats were examined in different experiments, with 293 SCI rats not receiving any therapeutic intervention (except normal saline or PBS), 78 rats receiving synthetic scaffold-assisted cell transplantation, and 219 rats receiving natural scaffold-assisted cell transplantation. For the study participants, the selected animal models were all rats, and the methods of causing spinal cord injury included contusion (32%), spinal cord hemisection (40%), and spinal cord transection (28%). Regarding intervention, the cell types used for cell transplantation were primarily mesenchymal stem cells (MSCs) and neural stem cells, with a total number of cells transplanted ranging from 10,000 to 5000000 and a time span from injury to therapeutic intervention ranging from immediately to 35 days. For comparison, the vehicle group was used as the control group, while the cell transplantation combined scaffold served as the experimental group. The components of natural scaffolds include collagen, fibroin, acellular, fibrin, and sodium alginate. The synthetic scaffolds were mainly synthetic polymers, such as polylactic-co-glycolic acid (PLGA). In terms of outcomes, the duration of the assessment was all greater than 4 weeks, ranging from 4 weeks to 2 years. The BBB score was used to measure the functionality improvement of the hind limbs, and two or more independent researchers evaluated 80% of the studies to avoid human-induced error (Table 2).

**Table 2:** Summary of included studies

Author (year)	Gender; strain; species; weight	Model, location and severity of injury	Scaffold	Cell donor; cell source transplant type	Injury to trans-plant interval (days)	Number of trans planted cell	Type of graft	Immune-suppressive; antibiotic; blinding	Follow-up (weeks)
Wang (2020)	Female; SD rat; 200–220	Contusion; T10; severe	Matrigel scaffold	Rat; brain; IS	7	5 × 10^5^	Allograft	No; yes; yes	8
Wang (2017)	Male; SD rat; 200	Hemisection; T9–T10; severe	Acellular spinal cord scaffold	Rat; bone marrow; IS	0	NR	Allograft	No; yes; yes	8
Kim (2016)	Male; SD rat; 282–322	Contusion; T9; severe	PLGA or chitosan scaffold	Rat; bone marrow; IS	0	1 × 10^6^	Allograft	No; yes; yes	6
You (2019)	Male; SD rat; 200–240	Contusion; T9; severe	Porous silk fibroin scaffold	Rat; bone marrow; IS	0	1 × 10^6^	Allograft	No; no; yes	4
Liu (2022)	Female; SD rat; 200–250	Transection; T8–T9; severe	Sodium alginate/gelatin scaffold	Rat; brain; IS	0	2 × 10^4^	Allograft	No; yes; yes	8
Hosseini (2016)	Male; SD rat; 250–300	Contusion; T7; severe	Alginate scaffold	Rat; brain; IS	1	1 × 10^5^	Allograft	No; no; yes	12
Wang (2016)	Female; SD rat; 250–300	Hemisection; T11; severe	Silk fibroin scaffold	Human; amniotic membrane; IS	7	1 × 10^6^	Xenograft	No; yes; no	4
Jiao (2017)	Female; Wistar rat; 240–260	Contusion; T8, T10; severe	The SF/AGs/GDNF scaffold	Human; umbilical cord; IS	0	1 × 10^6^	Xenograft	No; no; no	8
Peng (2018)	Male; SD rat; 223–277	Hemisection; T9; severe	Collagen scaffold	Rat; bone marrow; IS	0	1 × 10^6^	Allograft	No; no; yes	8
Chen (2014)	Male; SD rat; 200–250	Hemisection; T9–T10; severe	ASC Scaffold	Rat; bone marrow; IS	0	5 × 10^5^	Allograft	No; yes; yes	8
Liu (2013a)	Male; SD rat; 200–250	Hemisection; T9–T10; severe	ASC scaffold	Human; umbilical cord blood; IS	0	5 × 10^6^	Xenograft	No; yes; yes	8
Liu (2013b)	Female; SD rat; 280–300	Transection; T10; severe	Fibrin scaffold	Rat; nasal respiratory mucosa; IS	0	5 × 10^4^	Allograft	No; yes; yes	12
Marchini (2019)	Female; SD rat; 225	Hemisection; T9–T10; severe	SAP	Human; brain; IS	7	1.8 × 10^6^	Xenograft	Yes; yes; no	8
Zaminy (2013)	Male; Wistar rat; 250–300	Hemisection; T9–T10; severe	Atelocollagen honeycomb scaffold	Rat; bone marrow; IS	0	1 × 10^5^	Allograft	No; yes; no	8
Zarei-Kheirabadi (2020)	Male; Wistar rat; 250–280	Contusion; T10–T11; severe	HA-based hydrogel	Human; embryo; IS	7	1 × 10^6^	Xenograft	Yes; yes; yes	7
Hatami (2009)	Male; Wistar rat; 200–280	Hemisection; T10; severe	Collagen scaffold	Human; embryo; IS	7	3 × 10^5^	Xenograft	Yes; yes; yes	5
Wang (2021)	Male; SD rat; 200–230	Transection; T9–T11; severe	Nanofibrous silk fibroin scaffold	Rat; bone marrow; IS	0	5 × 10^5^	Allograft	No; yes; yes	8
Tavakol (2014)	Male; Wistar rat; 250-280	Contusion; T10; moderate	Matrigel	Human; endo-metrium; IS	10	1 × 10^4^	Xenograft	No; yes; no	6
Deng (2020)	Female; SD rat; 250–300	Transection; T10; severe	Collagen scaffold	Human; umbilical cord; IS	0	1 × 10^6^	Xenograft	No; yes; yes	8
Raynald (2019)	Female; SD rat; 200–250	Transection; T7–T9; severe	PPy/PLA nanofibrous scaffold	Rat; bone marrow; IS	0	1 × 10^5^	Allograft	No; yes; yes	6
Yang (2017)	Female; SD rat; 250–300	Transection; T10; severe;	PLGA	Rat; nerve/bone marrow; IS	0	1 × 10^5^	Allograft	No; yes; yes	8
Han (2019)	Female; SD rat; 220–250	Hemisection; T9–T10; severe	PLGA	Human; bone marrow; IS	0	1 × 10^5^	Xenograft	No; no; yes	4
Ropper (2017)	Female; NR rat; < 250	Hemisection; T9-T10; severe	PLGA	Human; bone marrow; IS	0	5 × 10^5^	Xenograft	Yes; no; yes	4
Liu (2015)	Female; SD rat; 220–250	Transection; T10; severe	PLGA-PEG	Mice; embryo; IS	0	1 × 10^6^	Xenograft	Yes; yes; yes	10
Hejĉl (2010)	Male; Wistar rat; 300–330	Contusion; T8–T9; severe	HPMA-RGD Hydrogel	Rat; bone marrow; IS	35	2 × 10^6^	Allograft	No; yes; yes	24

*IS* intra-spinal, NR not report, *PLGA* poly (latic-co-glycolic acid), *SF* silk fibroin, *GDNF* glial cell-derived neurotrophic factor, *AGs* alginates, *ASC* acellular spinal cord, *SAP* self-assembling peptides, *HA* hyaluronic acid, *PPy* polypyrrole, *PLA* polyactic acid, *PEG* polyethylene, *HPMA* N -(2-hydroxypropyl)-methacrylamide, *RGD* an attached oligopeptide sequence, *SD* Sprague-Dawley

### Quality Assessment of the Articles

Among the 25 pieces of literature included, most of the top 14 items of the included studies were considered low risk, indicating the good quality of the articles (Table 1). However, bladder expression was not documented in detail in six studies, and it was unclear whether the BBB score of hind limb movement in SCI rats was evaluated double blind in four studies. Furthermore, most studies did not explain in the literature why some animals were excluded (item 15).

### Evidence Network

**Fig. 2:**
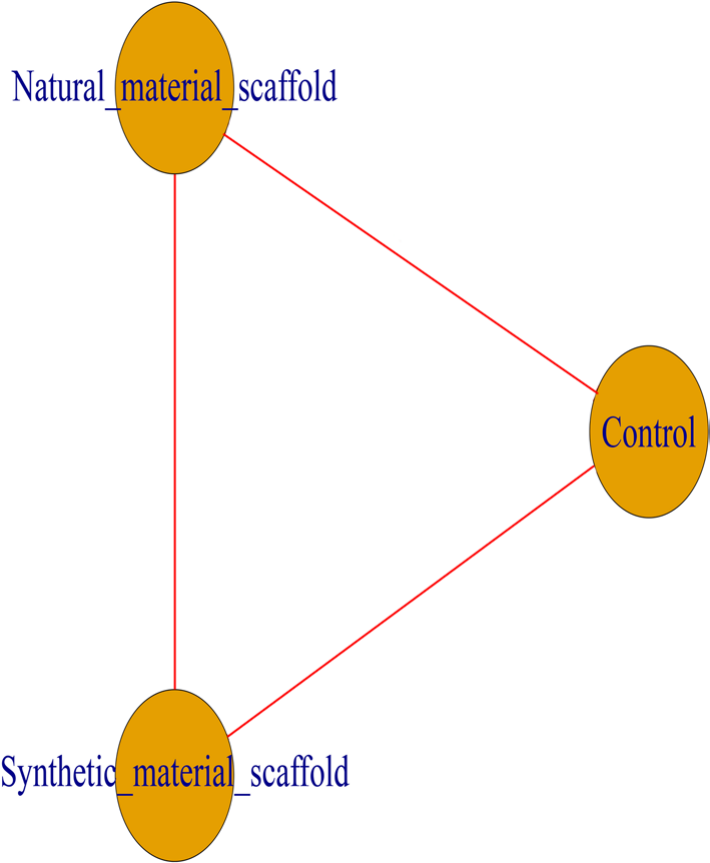
Network evidence map of different scaffold-assisted cell transplantation compared with vehicle control group

This NMA contains 19 articles comparing the efficacy of natural material scaffold-assisted cell transplantation and the vehicle control group in treating SCI rats, 7 articles on synthetic material scaffold-assisted cell transplantation, and a direct comparison of natural material against synthetic material scaffolds for the treatment of SCI rats. The red lines between each ball indicate that different interventions are directly compared. The width of the red line can be considered as the number of included studies (Fig. 2).

### Functional Outcomes

**Fig. 3:**
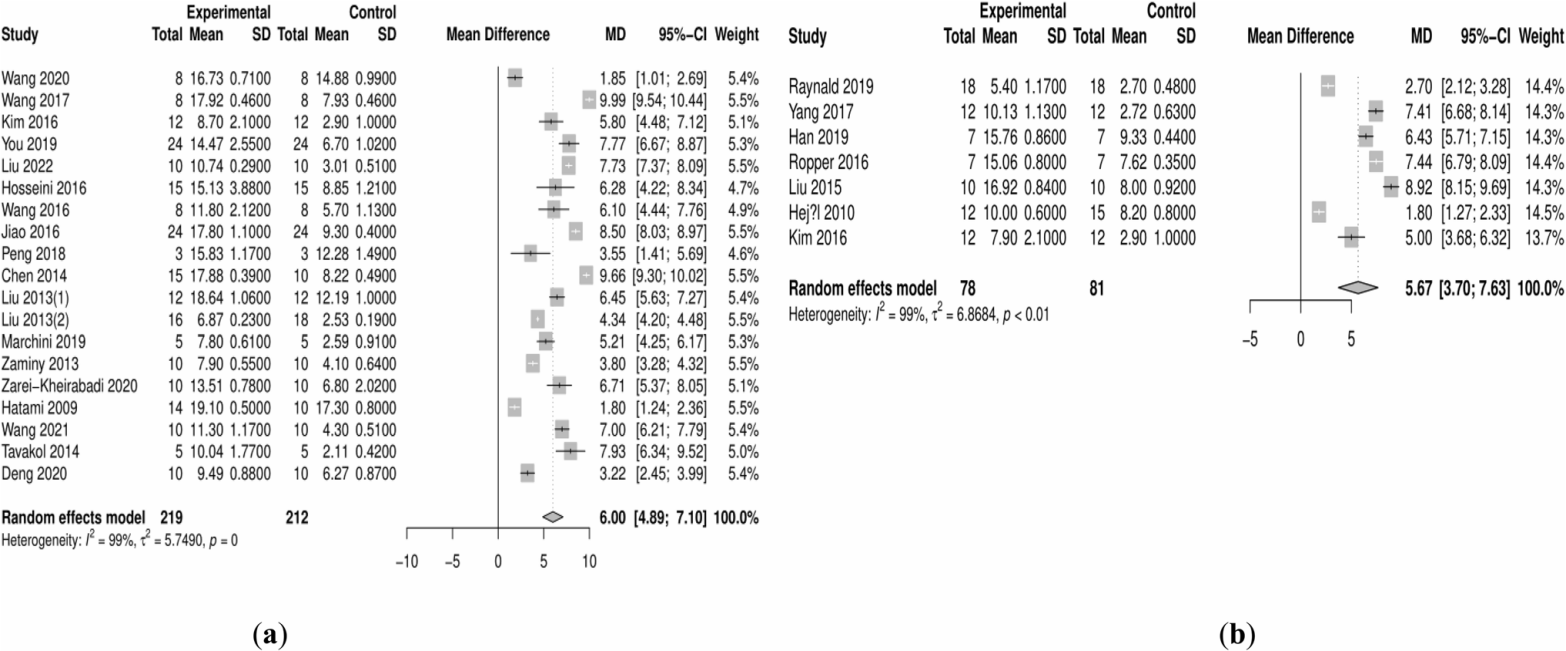
Forest plots of efficacy comparison between the experimental group (scaffold-driven cell transplantation) and vehicle control group with no treatment in SCI rats. **a** Natural scaffold; **b** synthetic scaffold

**Fig. 4:**
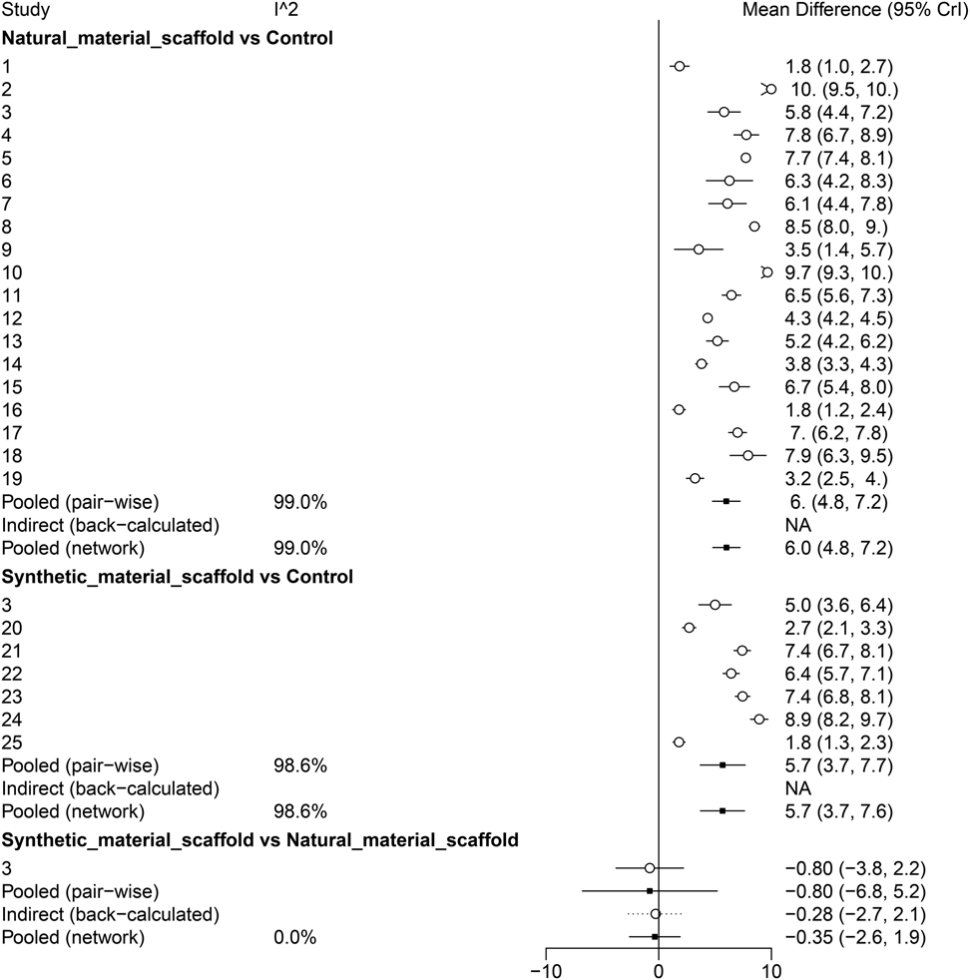
Forest plot of pairwise comparison between different interventions in SCI rats

Our results reveal that both natural scaffold-driven cell transplantation (MD: 6.0; 95% CI 4.8–7.2) and synthetic scaffold-driven cell transplantation (MD: 5.7; 95% CI 3.7–7.7) are superior to the control group in treating SCI rats, with statistically significant differences (Fig. 3). The direct comparison of two different types of scaffolds demonstrates that the synthetic scaffolds are inferior to the natural scaffolds (MD: -0.80; 95% CI -3.8 to 2.2), and the indirect comparisons also acknowledge subtle differences in the efficacy of synthetic materials (MD: -0.28; 95% CI -2.7 to 2.1). In summary, the NMA demonstrated that synthetic scaffold-assisted cell transplantation in SCI rats is less effective than natural materials (MD: -0.35; 95% CI -2.6 to 1.9). However, no statistical difference was observed between the two types of scaffold (Fig. 4).

### Subgroup Analysis

*I*^2^ was used to test for heterogeneity among the evidence (Fig. 4). Given that *I*^2^ was mostly over 50%, the heterogeneity among the evidence was high, so we tried to conduct a subgroup analysis to find the source of heterogeneity. Similarly, the random-effects model was applied to the meta-models.

Subgroup analysis was performed in two cohorts of natural and synthetic materials with respect to gender, mode of SCI injury, origin of cells, time period from injury to treatment, number of cells, whether immunosuppressants were used, whether antibiotics were used, duration of follow-up evaluation of BBB score, and whether the study is double blind or not (Tables 3, 4). Heterogeneity among subgroups of different categorical variables remains large; therefore, the source of heterogeneity between studies has not been tracked. Interestingly, significant differences were observed in terms of efficacy between subgroups with different cell counts in the natural material cohort (*P* < 0.01), whereas no other subgroup variables affected the difference in efficacy (all *P* > 0.05), suggesting that the number of cells transplanted is a potential factor influencing SCI treatment. To verify the results’ generalizability, we conducted a subgroup analysis that included cell transplantation with different scaffold types for SCI and its results supported the conclusions described above (Table 5, *P* < 0.01). However, in the synthetic cohort, cell type and the use of immunosuppressants become the differentiating variables for differences in efficacy (P < 0.01).

**Table 3:** Subgroup analysis of natural materials

Subgroups	Number of analysis	Effect size	Heterogeneity (*P* value)	*P* value (Inter-subgroup)
		MD (95% CI)		
Sex of rat	19	6.00 (4.89; 7.10)	99% (<0.001)	0.33
Male	12	6.42 (5.01; 7.83)	99% (< 0.01)	
Female	7	5.28 (3.50; 7.07)	99% (< 0.01)	
Injury model	19	6.00 (4.89; 7.10)	99% (< 0.001)	0.83
Contusion	7	6.39 (4.67; 8.12)	97% (< 0.01)	
Hemisection	8	5.86 (3.82; 7.89)	99% (< 0.01)	
Transection	4	5.58 (3.48; 7.67)	99% (< 0.01)	
Type of cell	19	6.00 (4.89; 7.10)	99% (< 0.001)	0.71
Mesenchymal stem cell	11	6.42 (4.96; 7.87)	99% (< 0.01)	
Neural stem cell	5	5.54 (3.48; 7.59)	98% (< 0.01)	
Others	3	5.21 (1.59; 8.83)	97% (< 0.01)	
Immunosuppressive	19	6.00 (4.89; 7.10)	99% (< 0.001)	0.27
Yes	3	4.53 (1.66; 7.39)	97% (< 0.01)	
No	16	6.27 (5.09; 7.46)	99% (< 0.001)	
Antibiotic	19	6.00 (4.89; 7.10)	99% (< 0.001)	0.48
Yes	15	5.83 (4.54; 7.12)	99% (< 0.001)	
No	4	6.71 (4.63; 8.79)	87% (< 0.01)	
Blinding	19	6.00 (4.89; 7.10)	99% (< 0.001)	0.73
Yes	14	5.89 (4.49; 7.28)	99% (< 0.001)	
No	5	6.28 (4.51; 8.05)	98% (< 0.01)	
Type of graft	19	6.00 (4.89; 7.10)	99% (< 0.001)	0.67
Allograft	11	6.20 (4.64; 7.76)	99% (< 0.01)	
Xenograft	8	5.70 (4.09; 7.32)	98% (< 0.01)	
Injury to transplant interval	19	6.00 (4.89; 7.10)	99% (< 0.001)	0.18
During first 24 h	13	6.52 (5.27; 7.76)	99% (< 0.01)	
7 days and over	6	4.86 (2.79; 6.93)	96% (< 0.01)	
Number of cells	19	6.00 (4.89; 7.10)	99% (< 0.001)	< 0.01*
Less than 5 × 10^5^	6	5.25 (3.29; 7.21)	99% (< 0.01)	
5 × 10^5^ and over	12	6.03 (4.72; 7.33)	98% (< 0.01)	
NR	1	9.99 (9.54; 10.44)	NA	
Follow-up duration	19	6.00 (4.89; 7.10)	99% (< 0.001)	0.97
8 weeks and over	13	6.00 (4.59; 7.42)	99% (< 0.001)	
Less than 8 weeks	6	5.96 (4.09; 7.84)	97% (< 0.01)	

*CI* confidence interval, *MD* mean difference, *NA* not available

*With statistical significance

**Table 4:** Subgroup analysis of synthetic materials

Subgroups	Number of analysis	Effect size	Heterogeneity (*P* value)	*P* value (Inter-subgroup)
		MD (95% CI)		
Sex of rat	7	5.67 (3.70; 7.63)	99% (< 0.01)	0.09
Male	2	3.34 (0.21; 6.47)	95% (< 0.01)	
Female	5	6.57 (4.51; 8.63)	98% (< 0.01)	
Injury model	7	5.67 (3.70; 7.63)	99% (< 0.01)	0.10
Contusion	2	3.34 (0.21; 6.47)	95% (< 0.01)	
Hemisection	2	6.95 (5.96; 7.94)	76% (0.04)	
Transection	3	6.34 (2.66; 10.01)	99% (< 0.01)	
Type of cell	7	5.67 (3.70; 7.63)	99% (< 0.01)	< 0.01*
Mesenchymal stem cell	6	5.12 (3.17; 7.08)	98% (< 0.01)	
Neural stem cell	1	8.92 (8.15; 9.69)	NA	
Immunosuppressive	7	5.67 (3.70; 7.63)	99% (< 0.01)	< 0.01*
Yes	2	8.16 (6.71; 9.61)	88% (< 0.01)	
No	5	4.65 (2.54; 6.77)	98% (< 0.01)	
Antibiotic	7	5.67 (3.70; 7.63)	99% (< 0.01)	0.22
Yes	5	5.16 (2.49; 7.83)	99% (< 0.01)	
No	2	6.95 (5.96; 7.94)	76% (0.04)	
Blinding	7	5.67 (3.70; 7.63)	99% (< 0.01)	NA
Yes	7	5.67 (3.70; 7.63)	99% (< 0.01)	
No	0	NA	NA	
Type of graft	7	5.67 (3.70; 7.63)	99% (< 0.01)	0.22
Allograft	5	5.16 (2.49; 7.83)	99% (< 0.01)	
Xenograft	2	6.95 (5.96; 7.94)	76% (0.04)	
Injury to transplant interval	7	5.67 (3.70; 7.63)	99% (< 0.01)	NA
During first 24 h	6	6.32 (4.55; 8.09)	98% (< 0.01)	
7 days and over	1	1.80 (1.27; 2.33)	NA	
Number of cells	7	5.67 (3.70; 7.63)	99% (< 0.01)	0.89
Less than 5 × 10^5^	3	5.51 (2.69; 8.32)	98% (< 0.01)	
5 × 10^5^ and over	4	5.79 (2.71; 8.87)	99% (< 0.01)	
Follow-up duration	7	5.67 (3.70; 7.63)	99% (< 0.01)	0.79
8 weeks and over	3	6.04 (1.78; 10.29)	99% (< 0.01)	
Less than 8 weeks	4	5.40 (3.34; 7.45)	98% (< 0.01)	

*CI* confidence interval, *MD* mean difference, *NA* not available

*With statistical significance

**Table 5:** Subgroup analysis of natural and synthetic materials

Subgroups	Number of analysis	Effect size	Heterogeneity (*P* value)	*P* value (Inter-subgroup)
		MD (95% CI)		
Sex of rat	25	5.94 (4.96; 6.92)	99% (< 0.001)	0.82
Male	13	6.05 (4.57; 7.53)	99% (< 0.01)	
Female	12	5.82 (4.48; 7.16)	99% (< 0.01)	
Injury model	25	5.94 (4.96; 6.92)	99% (< 0.001)	0.97
Contusion	8	5.80 (3.92; 7.67)	98% (< 0.01)	
Hemisection	10	6.08 (4.45; 7.72)	99% (< 0.01)	
Transection	7	5.90 (4.09; 7.71)	99% (< 0.01)	
Type of cell	25	5.94 (4.96; 6.92)	99% (< 0.001)	0.91
Mesenchymal stem cell	16	6.01 (4.78; 7.25)	99% (< 0.001)	
Neural stem cell	6	6.12 (4.11; 8.13)	97% (< 0.01)	
Others	3	5.21 (1.59; 8.83)	97% (< 0.01)	
Immunosuppressive	25	5.94 (4.96; 6.92)	99% (< 0.001)	0.95
Yes	5	6.01 (3.60; 8.41)	99% (< 0.01)	
No	20	5.92 (4.82; 7.03)	99% (< 0.001)	
Antibiotic	25	5.94 (4.96; 6.92)	99% (< 0.001)	0.17
Yes	19	5.70 (4.49; 6.90)	99% (< 0.001)	
No	6	6.88 (5.67; 8.10)	88% (< 0.01)	
Blinding	25	5.94 (4.96; 6.92)	99% (< 0.001)	0.69
Yes	20	5.85 (4.69; 7.01)	99% (< 0.001)	
No	5	6.28 (4.51; 8.05)	98% (< 0.01)	
Type of graft	25	5.94 (4.96; 6.92)	99% (< 0.001)	0.98
Allograft	15	5.92 (4.51; 7.34)	99% (< 0.001)	
Xenograft	10	5.95 (4.63; 7.28)	98% (< 0.01)	
Injury to transplant interval	25	5.94 (4.96; 6.92)	99% (< 0.001)	0.24
During first 24 h	19	6.27 (5.18; 7.37)	99% (< 0.001)	
7 days and over	6	4.86 (2.79; 6.93)	96% (< 0.01)	
Number of cells	25	5.94 (4.96; 6.92)	99% (< 0.001)	< 0.01*
Less than 5 × 10^5^	9	5.33 (3.82; 6.84)	99% (< 0.01)	
5 × 10^5^ and over	15	6.02 (4.76; 7.29)	98% (< 0.01)	
NR	1	9.99 (9.54; 10.44)	NA	
Follow-up duration	25	5.94 (4.96; 6.92)	99% (< 0.001)	0.84
8 weeks and over	16	6.01 (4.68; 7.33)	99% (< 0.001)	
Less than 8 weeks	9	5.81 (4.34; 7.27)	97% (< 0.01)	

*CI* confidence interval, *MD* mean difference, *NA* not available

*With statistical significance

### Inconsistency Test

**Fig. 5:**
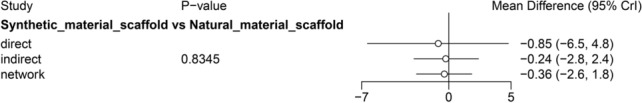
Inconsistency test forest map

The interventions in the NMA form a closed loop. Because consistency testing is required, the node-splitting method and its Bayes *P* value are used to evaluate the inconsistencies of our results between direct and indirect outcomes. In Fig. 5, although they are not statistically significant, the direct (MD: -0.85; 95% CI -6.5 to 4.8) and the indirect results (MD: -0.24; 95% CI -2.8 to 2.4) both support that natural materials are better than synthetic materials in SCI rats for cell transplantation. Similarly, the inconsistency test demonstrates that the *P* value (0.8345) is greater than 0.05, indicating there is no inconsistency in the overall analysis, and the results of direct comparison and indirect comparison are consistent (Fig. 5).

### Ranking Probability

**Fig. 6:**
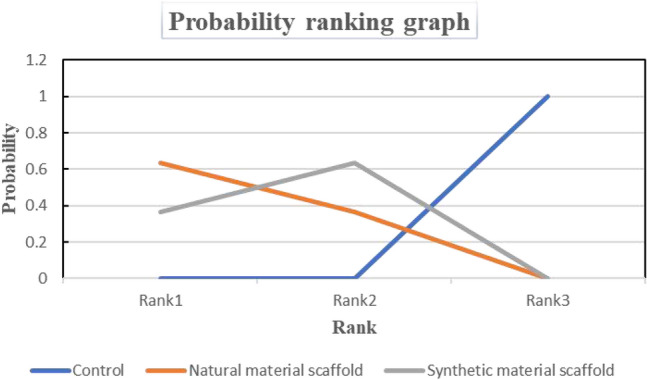
The efficacy ranking diagram of different interventions in the treatment of SCI rats

The ranking probabilities of these different interventions of the NMA are illustrated in Fig. 6. Ranking probability is used to evaluate the possibility of optimal treatment for SCI rats. According to the ranking probability map, the best probability order of the effect of two different scaffold-assisted cell transplantation on BBB score was natural material (probability = 0.62) > synthetic material (probability = 0.38) > vehicle group (probability = 0).

### Convergence Diagnosis

**Fig. 7:**
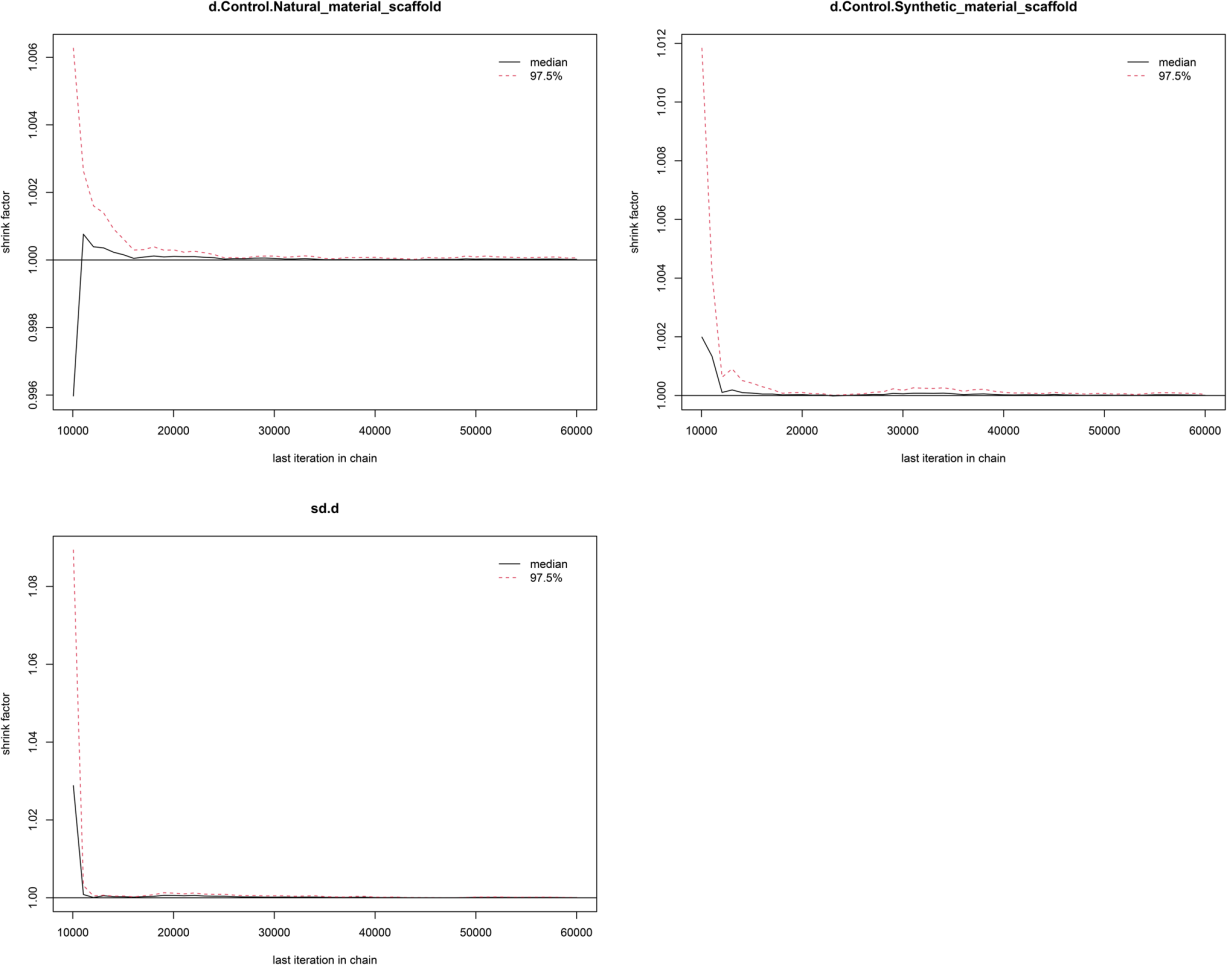
Convergence diagnostic graph

**Fig. 8:**
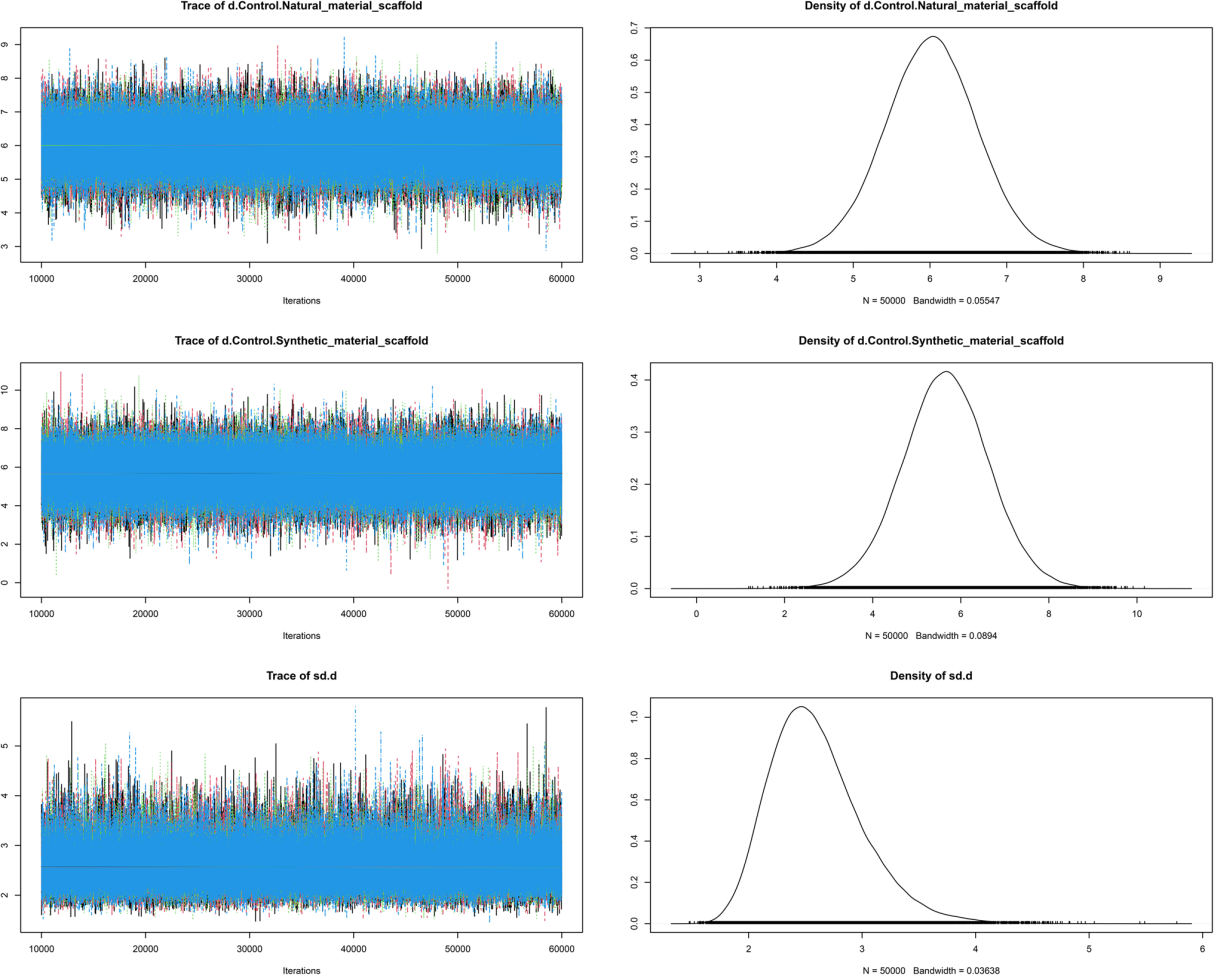
Trajectories and density maps

The convergence diagnostic graph is close to 1, indicating that the NMA model fits well (Fig. 7). The trajectory and density map of the included studies are visualized (Fig. 8), and the figure shows that the bandwidth value is close to 0, indicating good convergence.

### Publication Bias

**Fig. 9:**
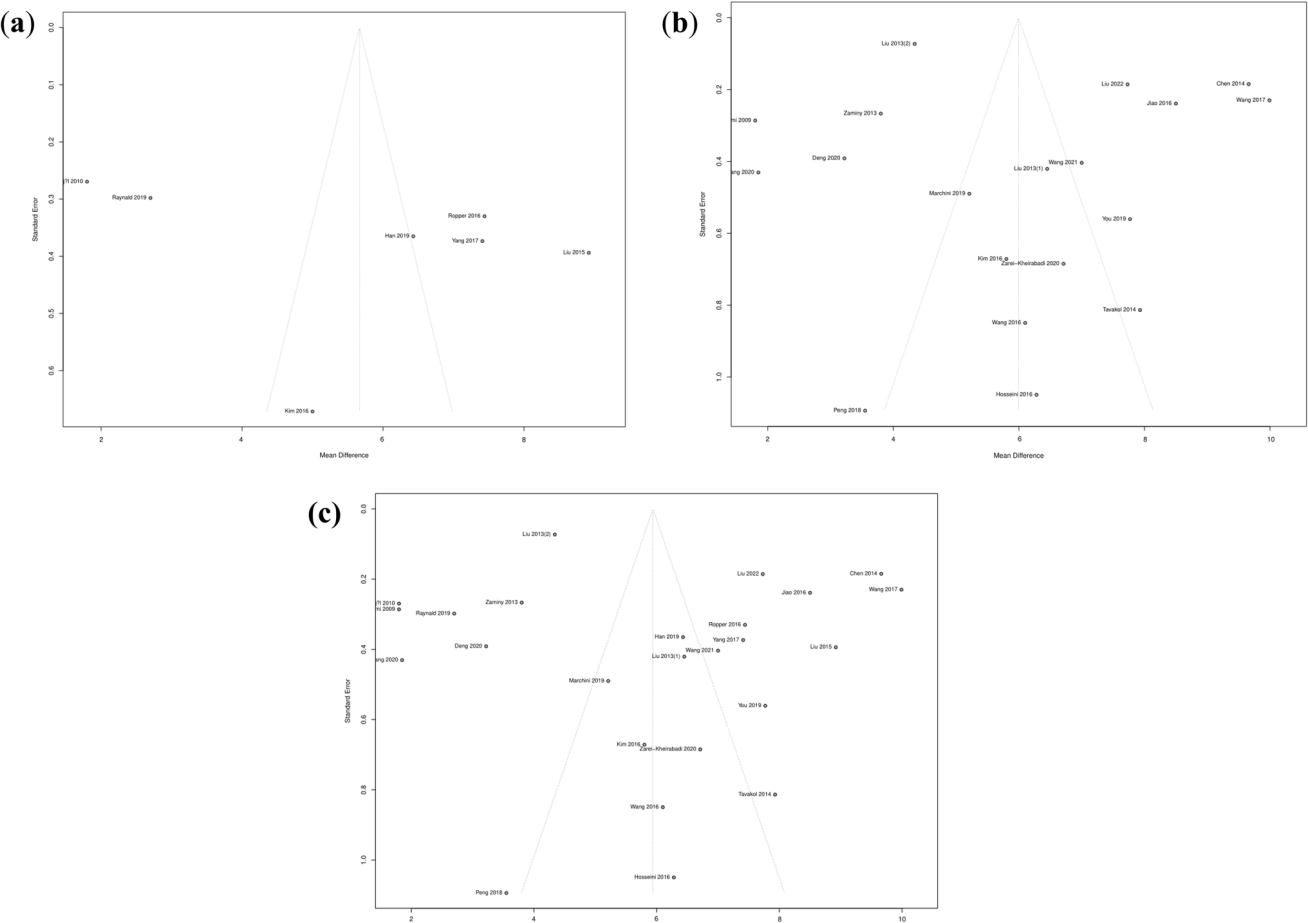
Publication bias funnel plot. **a** Study cohort of synthetic material scaffolds. **b** Study cohort of natural material scaffolds. **c** Total study cohort

Funnel plots are made from the synthetic material cohort, natural material cohort, and all the included literature. They are relatively symmetrical to the naked eye, indicating small publication bias (Fig. 9a–c).

## Discussion

Cell transplantation is reviewed as an emerging therapeutic strategy for improving motor function after SCI (Lv et al. 2021). However, the scaffold used as a carrier for cell transplantation has not been thoroughly investigated. Conducting a NMA is of critical importance to compare the efficacy of different types of scaffolds, which can provide reference value for subsequent clinical experiments and even large-scale clinical applications. From the perspective of evidence-based medicine, this study objectively evaluates the rehabilitation effect of cell transplantation on motor function after SCI in rats and compares the efficacy of two different types of scaffolds. In the included studies, the BBB score is used to evaluate the motor function of rats. The higher the score of rats within a certain period after different interventions, the better the prognosis. Our study reveals that cell transplantation can effectively restore the motor function of rats after SCI compared with the vehicle control group, and the natural scaffold seems to be better when considering the type of scaffolds assisted with cell transplantation.

First, our findings show that no matter what type of scaffold-driven cell transplantation is used, it can significantly improve limb movement recovery after SCI in rats when compared to the control group. This result is expected, given that the cell types used for cell transplantation are mostly stem cells known for continuous self-renewal and multidirectional differentiation. They can secrete a variety of nutritional factors to repair damaged tissues and recognize and differentiate into various neurons and glial cells. Based on these unique mechanisms, exogenously supplemented nerve regeneration therapy, that is, cell transplantation, may play a pivotal role in the treatment of SCI by replacing damaged cells, axons, and circuits in the spinal cord.

Second, our NMA results suggest that natural scaffolds are better as cell carriers in cell transplantation. This is reflected in two ways: first, as far as we know, there are very few studies on the treatment of SCI in terms of scaffold types, which highlights the importance of our meta-analysis. The NMA still includes a direct comparison of literature evidence. Direct evidence demonstrates that cell transplantation assisted by natural scaffolds is superior to that by synthetic scaffolds in terms of treating motor function in SCI rats. Moreover, the indirect comparison is completed by the vehicle control group of rats that acts as a transfer connection node, and the analysis results also support the use of natural scaffolds. Based on the above, the generalized consistency hypothesis is established, so the analysis results are of evidence-based significance. The reason for this difference in efficacy may be the poor cell compatibility of the synthetic scaffold, which is prone to the formation of an acidic environment during the degradation process, thus causing certain damage to the cells (Liu et al. 2019a, b). However, natural scaffolds have good biocompatibility, which is reflected in good biodegradability and low side effects. Notably, although the synthetic scaffold is slightly inferior to the natural scaffold in the functional prognosis of SCI rats after surgery, no statistical significance was observed, whether in the direct or indirect comparison of the two scaffold types. Therefore, many high-quality RCTs are warranted to verify our conclusions.

A previous meta-analysis found that acellular spinal cord scaffolds + MSCs were significantly superior to MSCs + other scaffolds when compared to vehicle controls; therefore, acellular spinal cord scaffolds as natural scaffolds strongly support our conclusions about the superiority of natural materials (Yousefifard et al. 2019). However, Yousefifard et al. classified scaffold types in a very specific and nuanced way, specific to the acellular spinal cord, with limited assistance from MSCs. When studies on different scaffolds for MSC transplantation are included in the analysis, our results also suggested that the acellular spinal cord is the optimal relative compared to other scaffolds (data were not present). It should be admitted that our research is relatively broad, aiming to provide a general direction of scaffold type for future research.

Given the broad nature of the NMA conclusions, it is important to recognize that some studies differ significantly, requiring further investigation. In this study, Participants: SCI rats caused by mainstream means; Intervention: rats received cell transplantation or cell transplantation therapy combined with different material scaffolds after SCI; Control or Comparison: the rats were injected saline or PBS equal to the amount of cell fluid after surgery of causing SCI; and Outcome: BBB score, an internationally accepted hindlimb motor function recovery score for rats. As with the PICOS (Participants, Intervention, Control, Outcome, Study design) principle, some details of the intervention (I) and function (O) are worth examining, and the rest of the direction details are almost the same between studies. However, our subgroup analysis of 10 factors did not accurately locate the source of heterogeneity because the number of animals included in the studies was too small to identify significant differentiating variables that led to high heterogeneity. Interestingly, in the subgroup analysis, the cell count was a statistically significant measure of efficacy in treating SCI rats. As a binary variable, 5 × 10^5^ is taken as a critical value of the number of transplanted cells, and we find that a value greater than 5 × 10^5^ is better for subsequent nerve regeneration and recovery in SCI rats. As previously reported, compared to low doses, high-dose transplanted cells can promote the differentiation of transplanted cells into neurons and migration to the injured distal end by regulating the expression of some neurotrophic factors to provide better nutritional support for the site of the spinal cord injury (Teng et al. 2001). Notably, the large number of cells is not always better (Piltti et al. 2017). When the number of stem cells reaches a certain level, more stem cells will lead to a lower proportion of differentiated oligodendrocytes, thus affecting the recovery efficacy, as reported in the meta-analysis by Yousefifard et al. (Yousefifard et al. 2019; Piltti et al. 2017). Their analysis displayed a parabolic trend in the effectiveness of cell dose in the treatment of SCI. Unexpectedly, the use of cell types and immunosuppressive agents can affect the prognosis in the synthetic scaffold cohort. In the natural materials cohort, mesenchymal stem cell is better at improving motor function in SCI rats, but there is no statistical significance. Recently, a systematic review of clinical cell transplantation shows that mesenchymal stem cell ranks first in most of the cells used for transplantation in improving the ASIA Impairment Scale (AIS) grade of SCI patients (Xu et al. 2022). However, only in terms of improvement of motor function, neural stem cell is better than MSC, which is consistent with the results of our synthetic material cohort. This cohort contains only seven studies, and the number of studies between subgroups is not evenly distributed, so this result should be interpreted with caution.

Regarding the optimal timing of cell transplantation, our study shows that no statistical significance in functional recovery was observed between acute (24 h after SCI) and subacute (within 7–10 days after SCI) cell transplantation on both natural and synthetic scaffolds, despite the fact that the acute phase is better. In terms of the transplantation time, some studies reported that the acute phase of SCI has a high rate of apoptosis and inflammatory reactions, resulting in the formation of a toxic microenvironment at the site of spinal cord injury, so glucocorticoid shock therapy is given as a treatment option in the acute phase, and precious cells should be transplanted in the subacute phase (Oh and Jeon 2016). However, our results are similar to those of prior studies; thus, we suggest that transplantation in the acute stage will have better results (Yousefifard et al. 2019). This may be attributed to the fact that secondary damage of SCI typically occurs within a week. Early transplantation of stem cells can effectively mitigate the occurrence of secondary damage and facilitate the restoration of neurological function (Rouanet et al. 2017). Chronic spinal cord injury has many disadvantages in restoring nerve function, including apoptotic neurons in the injured area and surrounding scar tissue, both of which form cavitation that greatly inhibits axon regeneration (Silver and Miller 2004; Hashimoto et al. 2024). In animal studies, cell transplantation combined with scaffolds has also been reported to be effective in chronic SCI with cavity formation, regardless of incomplete or complete SCI injury (Hashimoto et al. 2023; Nori et al. 2018; Liu et al. 2019a, b). Similarly, scaffold is also used clinically, which can improve sensory or motor function in varying proportions in patients with chronic complete SCI (Tang et al. 2022). Therefore, scaffold combined therapy may become a new trend in patients with chronic SCI. However, given that the studies we included did not evaluate enough chronic SCI rats, we cannot draw a firm conclusion about the efficacy of different material scaffold in chronic SCI from our study, which will be an interesting topic for research to address.

One of the advantages of our study is that the subjects included in the study are all rats. We excluded studies on monkeys, dogs, and other animals during the inclusion process to avoid the analytical bias caused by species differences as much as possible. Moreover, we searched many databases to incorporate sufficient literature to avoid publication bias. Because the included literature not only used contusion or complete transection intervention to cause complete SCI but also used spinal cord hemisection to make incomplete SCI in rats, our included studies are universal in the SCI types of various severity. However, limitations should be concerned. Firstly, many of the rat models of spinal cord injury used in these included studies differ from clinical conditions, such as transection and hemisection (Sharif-Alhoseini et al. 2017). Secondly, it is should be admitted the BBB score is subjective (Basso et al. 1995), but the vast majority of the studies we included were double blind in their functional assessment, so the shortcomings of subjectivity can be avoided. Finally, there is still considerable heterogeneity between studies, so the results need to be treated critically. It should be emphasized that there are few studies on the functional recovery of chronic SCI for combined treatment, so it is recommended that subsequent clinical trials focus on acute spinal cord injury (Hejcl et al. 2010). Large-scale, high-quality animal model experiments are warranted to validate our results.

## Conclusion

Natural scaffolds and synthetic scaffolds are equally effective in cell transplantation of SCI rats without significant differences. In the future, the findings need to be validated in multicenter, large-scale RCTs in clinical practice.

## Supplementary Information

Below is the link to the electronic supplementary material.

Supplementary file1 (DOCX 16 KB)

## Data Availability

The data on rats was obtained from the published literature after searching databases; no available data exist.
