# Supplementary material for: The Efficacy of Different Material Scaffold-Guided Cell Transplantation in the Treatment of Spinal Cord Injury in Rats: A Systematic Review and Network Meta-analysis
Source: Cell Mol Neurobiol. 2024 May 4;44:43. doi: 10.1007/s10571-024-01465-6 (PMC11069479; doi:10.1007/s10571-024-01465-6)
Supplement: Supplementary file 1 — Supplementary file1 (DOCX 16 KB) [file 10571_2024_1465_MOESM1_ESM.docx]

**Supplementary material**

Search strategy for seeking relevant studies.

**Pubmed**

1- ((((((((((((((((Spinal Cord Injury[MeSH Terms]) OR (Spinal Cord Injuries)) OR (Spinal Cord Trauma)) OR (Spinal Cord Traumas)) OR (Traumatic Myelopathies)) OR (Traumatic Myelopathy)) OR (Injured Spinal Cord)) OR (Spinal Cord Transection)) OR (Spinal Cord Transections)) OR (Spinal Cord Laceration)) OR (Spinal Cord Lacerations)) OR (Post-Traumatic Myelopathies)) OR (Post-Traumatic Myelopathy)) OR (Spinal Cord Contusion)) OR (Spinal Cord Contusions)) OR (Spinal Cord Compression)) OR (Spinal Cord Hemisection)

2- (Cell Transplantation[MeSH Terms]) OR (Cell Transplantations)

3- (Stem Cell Transplantation[MeSH Terms]) OR (Stem Cell Transplantations)

4- (((Tissue Scaffold[MeSH Terms]) OR (Tissue Scaffolds)) OR (Tissue Scaffolding)) OR (Tissue Scaffoldings)

#1 AND (#2 OR #3) AND #4

**Embase**

1. 'spinal cord injury'/exp OR 'spinal cord injuries'/exp OR 'spinal cord trauma'/exp OR 'spinal cord traumas'/exp OR 'traumatic myelopathies'/exp OR 'traumatic myelopathy'/exp OR 'injured spinal cord'/exp OR 'spinal cord transection'/exp OR 'spinal cord transections'/exp OR 'spinal cord laceration'/exp OR 'spinal cord lacerations'/exp OR 'post-traumatic myelopathy'/exp OR 'post-traumatic myelopathies'/exp OR 'spinal cord contusion'/exp OR 'spinal cord contusions'/exp OR 'spinal cord compression'/exp OR 'spinal cord hemisection'/exp
2. 'cell transplantation'/exp OR 'cell transplantations'/exp
3. 'stem cell transplantation'/exp OR 'stem cell transplantations'
4. 'tissue scaffold'/exp OR 'tissue scaffolds'/exp' OR 'tissue scaffolding'/exp OR 'tissue scaffoldings '/exp

#1 AND (#2 OR #3) AND #4

**Web of Science**

1- TS=(“Spinal Cord Injury” OR “Spinal Cord Injuries” OR “Spinal Cord Trauma” OR “Spinal Cord Traumas” OR “Traumatic Myelopathy” OR “Traumatic Myelopathies” OR “Injured Spinal Cord” OR “Spinal Cord Transection” OR “Spinal Cord Transections” OR “Spinal Cord Laceration” OR “Spinal Cord Lacerations” OR “Post-Traumatic Myelopathy” OR “Post-Traumatic Myelopathies” OR “Spinal Cord Contusion” OR “Spinal Cord Contusions” OR “Spinal Cord Compression” OR “Spinal Cord Hemisection” )

2- TS=(“Cell Transplantation” OR “Cell Transplantations”)

3- TS=(“Stem Cell Transplantation” OR “Stem Cell Transplantations”)

4- TS=(“Tissue Scaffold” OR “Tissue Scaffolds” OR “Tissue Scaffolding” OR “Tissue Scaffoldings”)

#1 AND (#2 OR #3) AND #4

**Cochrane Library**

1- “Spinal Cord Injury” OR “Spinal Cord Injuries” OR “Spinal Cord Trauma” OR “Spinal Cord Traumas” OR “Traumatic Myelopathy” OR “Traumatic Myelopathies” OR “Injured Spinal Cord” OR “Spinal Cord Transection” OR “Spinal Cord Transections” OR “Spinal Cord Laceration” OR “Spinal Cord Lacerations” OR “Post-Traumatic Myelopathy” OR “Post-Traumatic Myelopathies” OR “Spinal Cord Contusion” OR “Spinal Cord Contusions” OR “Spinal Cord Compression” OR “Spinal Cord Hemisection”

2- “Cell Transplantation” OR “Cell Transplantations”

3- “Stem Cell Transplantation” OR “Stem Cell Transplantations”

4- “Tissue Scaffold” OR “Tissue Scaffolds” OR “Tissue Scaffolding” OR “Tissue Scaffoldings”

#1 AND (#2 OR #3) AND #4 :ti,ab,kw
